# Structure cristalline de type alluaudite K_0.4_Na_3.6_Co(MoO_4_)_3_

**DOI:** 10.1107/S2056989014025894

**Published:** 2015-01-01

**Authors:** Rawia Nasri, Noura Fakhar Bourguiba, Mohamed Faouzi Zid

**Affiliations:** a Laboratoire de Matériaux et Cristallochimie, Faculté des Sciences de Tunis, Université de Tunis El Manar, 2092 El Manar Tunis, Tunisie

**Keywords:** crystal structure, triple molybdate, alluaudite-type

## Abstract

A new triple molybdate, potassium sodium cobalt tris­(molybdate), K_0.4_Na_3.6_Co(MoO_4_)_3_, was synthesized using solid-state reactions. The Co^2+^ and one Na^+^ cation are located at the same general site, each with occupancy 0.5. Another site (site symmetry 2) is occupied by Na^+^ and K^+^ cations, with occupancies of 0.597 (7) and 0.402 (6), respectively. The other two Na^+^ cations and one of the two Mo atoms lie on special positions (site symmetries -1, 2 and 2, respectively). The structure is characterized by *M*_2_O_10_ (*M* = Co/Na) dimers, which are linked by MoO_4_ tetra­hedra, forming infinite layers. The latter are connected firstly by insertion of one type of MoO_4_ tetra­hedra and secondly by sharing corners with the other type of MoO_4_ tetra­hedra. This results in an open three-dimensional framework with the cavities occupied by the Na^+^ and K^+^ cations. The structure is isotypic with Na_3_In_2_As_3_O_12_ and Na_3_In_2_P_3_O_12_. A comparison is made with structures such as K_2_Co_2_(MoO_4_)_3_ and *β*-NaFe_2_(MoO_4_)_3_ and their differences are discussed.

## Abstract 2

A new triple molybdate K_0.4_Na_3.6_Co(MoO_4_)_3_ was synthesized using solid-state reaction at 973 K and characterized by X-ray diffraction. The structure is characterized by *M*_2_O_10_ (*M* = Co/Na) dimers, which are linked by MoO_4_ tetra­hedra, forming infinite layers. The latter are connected on one hand by insertion of Mo1O_4_ tetra­hedra and secondly by sharing corners with Mo2O_4_ tetra­hedra.

## Contexte chimique   

L’assemblage octa­èdres-tétraèdres dans les matériaux inorganiques conduit à des charpentes ouvertes présentant des propriétés physiques importantes, en particulier la conduction ionique (Judeinstein *et al.*, 1994 ▸; Sanz *et al.*, 1999 ▸). La richesse structurale dans ces matériaux nous a encouragé de faire l’exploration des systèmes *A*–Co–Mo–O (*A* = ion monovalent). La synthèse conduit à un nouveau matériau de formulation K_0.4_Na_3.6_Co(MoO_4_)_3_ appartenant à la famille des alluaudites (Moore, 1971 ▸; Yakubovich *et al.*, 2005 ▸; Hatert, 2008 ▸). Un examen bibliographique montre que le matériau étudié est isostructural aux composés: Na_3_In_2_As_3_O_12_ et Na_3_In_2_P_3_O_12_ (Lii & Ye, 1997 ▸).

## Commentaire structurelle   

**Figure 1:**
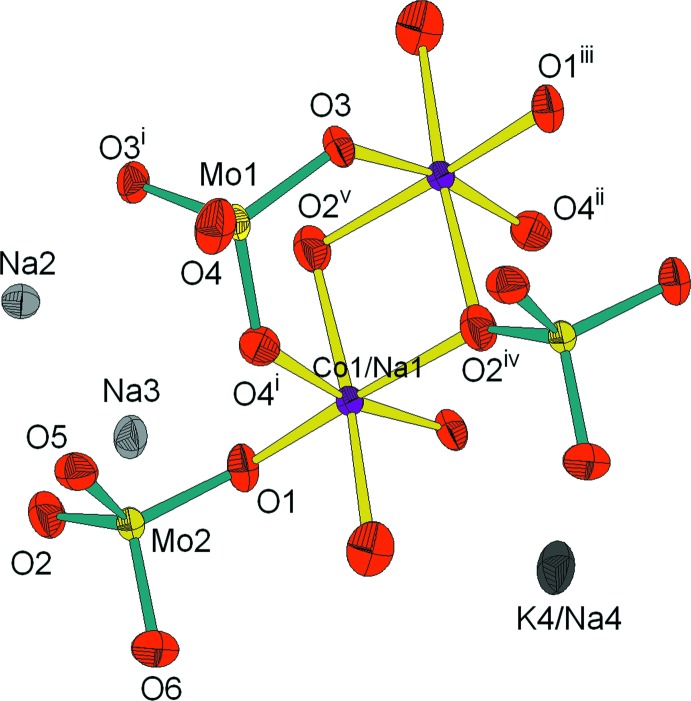
Unité asymétrique dans K_0.4_Na_3.6_Co(MoO_4_)_3_. Les éllipsoïdes ont été définis avec 50% de probabilité. [Code de symétrie: (i) −*x* + 1, *y*, −*z* + 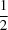; (ii) *x*, −*y* + 1, *z* − 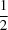; (iii) −*x* + 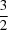, *y* − 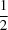, −*z* + 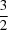; (iv) −*x* + 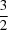, −*y* + 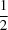, −*z* + 1; (v) *x*, −*y* + 1, *z* + 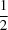.

L’unité asymétrique referme un dim­ère *M*_2_O_10_ (*M* = Co/Na) connecté par mise en commun de sommets avec deux tétraèdres et deux tétraèdres Mo2O_4_ différents et par partage d’arête avec un tétraèdres Mo1O_4_. La compensation de charge dans la structure est assurée par les cations Na^+^ et K^+^ (Fig. 1 ▸).

**Figure 2:**
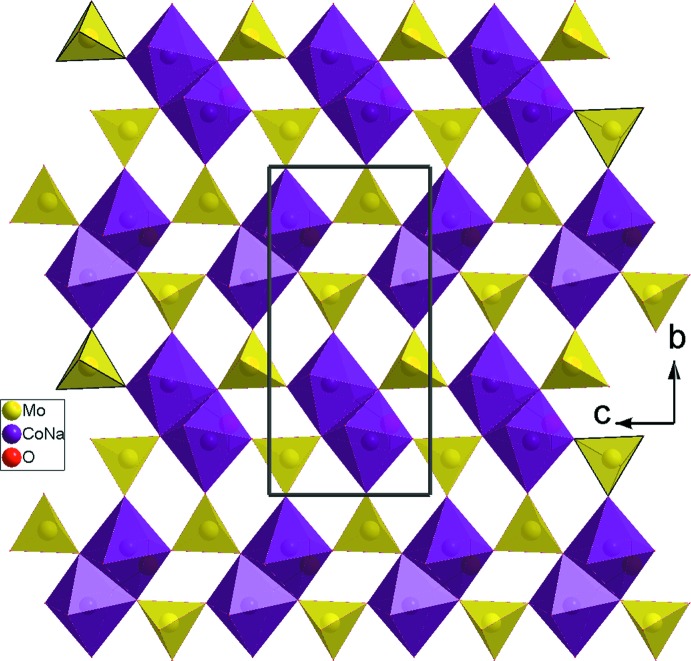
Projection d’une couche disposée parallèlement au plan (100).

Dans la charpente anionique deux octa­èdres juxtaposés, se lient par partage d’arête pour former des dimères *M*_2_O_10_ (*M* = Co1/Na1). Ces derniers se connectent par ponts triples avec les tétraèdres Mo2O_4_ pour donner les unités *M*_2_Mo_2_O_16_. Ces dernières se connectent à six autres unités identiques par mise en commun de sommets pour conduire à des couches infinies de type *M*_2_Mo_3_O_12_ (*M* = Co1/Na1) (Fig. 2 ▸).

**Figure 3:**
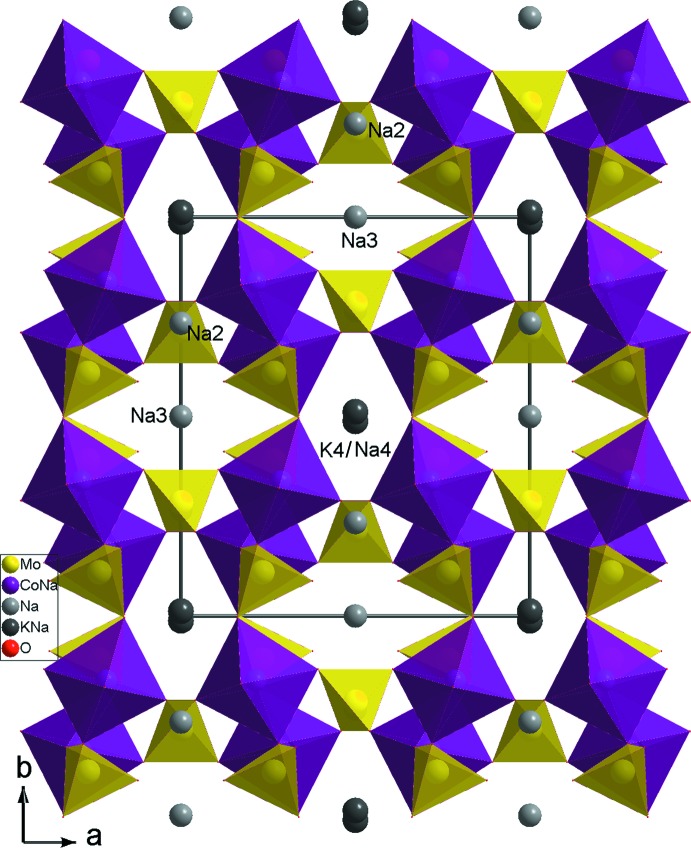
Projection de la structure de K_0.4_Na_3.6_Co(MoO_4_)_3_ selon *c*.

Deux couches adjacentes se lient, d’une part par insertion des tétraèdres Mo1O_4_ entre les couches et d’autre part par partage de sommets pour conduire à une charpente tri­dimensionnelle, possédant deux types de canaux larges, à section hexa­gonale, parallèles à l’axe *c* où logent les cations Na3 et (K4/Na4) (Fig. 3 ▸).

**Figure 4:**
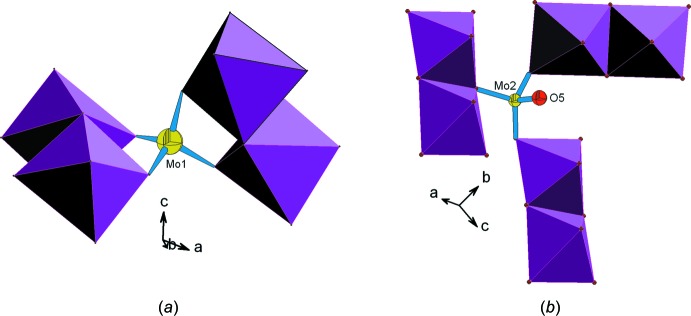
Représentation des environnements des tétraèdres: (*a*) Mo1O_4_, (*b*) Mo2O_4_.

Dans la charpente anionique chaque tétraèdre Mo1O_4_ partage ses quatre sommets avec respectivement quatre octa­èdres formant deux dimères appartenant à deux couches adjacentes (Fig. 4 ▸ *a*). Par contre, dans la structure chaque tétraèdre Mo2O_4_ met en commun seulement trois de ses sommets avec respectivement trois dimères différents appart­enant à la même couche, le quatrième sommet oxygène restant libre forme le groupement molybdyl [*d*(*M*=O) = 1,744 (2) Å] (Fig. 4 ▸ *b*). Un examen des caractéristiques géométriques relevées de l’étude structurale montre que les distances moyennes dans les tétraèdres MoO_4_ et dans les octa­èdres MO_6_ (*M* = Co1/Na1), sont égales respectivement à 1,761 (3) et 2,210 (3) Å. La première Mo–O, est conforme à celles rencontrées dans la bibliographie (Souilem *et al.*, 2014 ▸; Ennajeh *et al.*, 2013 ▸; Engel *et al.*, 2009 ▸). La seconde *M*—O (*M* = Co1/Na1), s’avère une moyenne entre celles Co—O (Engel *et al.*, 2009 ▸; Marzouki *et al.*, 2013 ▸) et Na—O (Muessig *et al.*, 2003 ▸; Baies *et al.*, 2006 ▸). De plus, le calcul des charges des ions, utilisant la formule empirique de Brown (Brown & Altermatt, 1985 ▸), conduit aux valeurs des charges des ions suivants: Mo1 (5,943), Mo2 (5,946), Co1/Na1 (1,822), Na2 (1,093), Na3 (0,838), K4/Na4 (0,868). La structure étudiée étant de type alluaudite, elle est similaire à celles rencontrées dans la littérature (Haj Abdallah & Haddad, 2008 ▸; Zid *et al.*, 2005 ▸). Elles diffèrent seulement par l’occupation des sites cation­iques. En effet, dans le composé Na_1.72_Mn_3.28_(AsO_4_)_3_ (Ayed *et al.*, 2002 ▸), le site cristallographique (1/2,*y*,3/4) du groupe d’espace *C*2/*c*, est occupé par l’atome de manganèse Mn1, contrairement à notre phase où ce site est occupé par l’alcalin Na2 (Fig. 3 ▸).

## Enquête de base de données   

**Figure 5:**
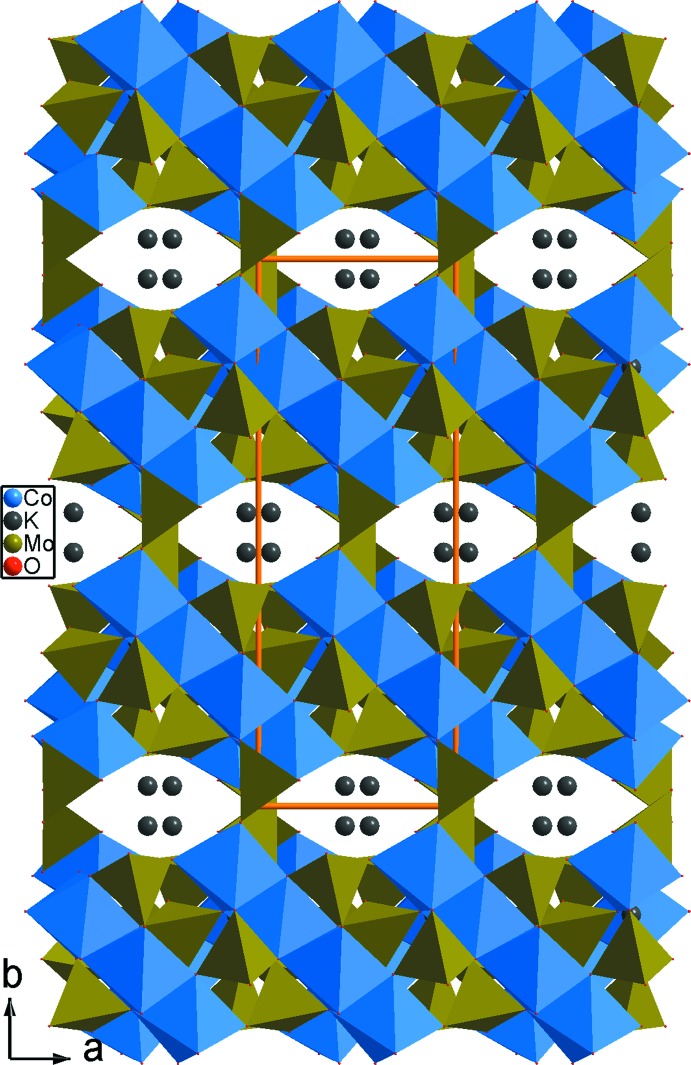
Projection de la structure de K_2_Co_2_(MoO_4_)_3_, selon *c*.

**Figure 6:**
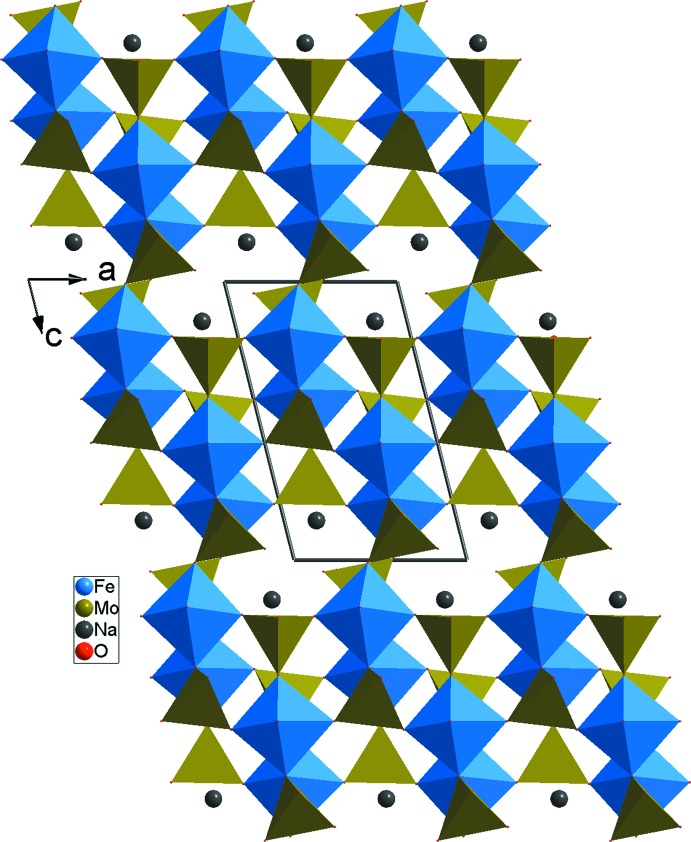
Projection de la structure de la variété *β*-NaFe_2_(MoO_4_)_3_, selon *b*.

Le composé de formulation K_2_Co_2_(MoO_4_)_3_ (Engel *et al.*, 2009 ▸), présente trois variétés allotropiques. Elles cristallisent dans le systéme monoclinique, groupes d’espace *P*2_1_/*c* ou bien *C*2/*c*. La charpente anionique dans K_2_Co_2_(MoO_4_)_3_ présente contrairement à notre structure des tétramères (Fig. 5 ▸) au lieu des dimères. L’association, par partage de sommets, des tétramères avec tous les tétraèdres MoO_4_ dans K_2_Co_2_(MoO_4_)_3_ conduit vers une structure bidimensionnelle. La comparaison de notre structure avec celle de la variété *β*-NaFe_2_(MoO_4_)_3_ (Muessig *et al.*, 2003 ▸), montre une différence dans la disposition des dimères *M*_2_O_10_ (*M* = Co/Fe). En effet dans la structure *β*-NaFe_2_(MoO_4_)_3_ les dimères sont orientés de la même façon. Leur association par partage de sommets avec les tétraèdres MoO_4_ conduit vers une charpente ouverte possédant de larges canaux où résident les cations Na^+^ (Fig. 6 ▸). Contrairement à notre matériau K_0.4_Na_3.6_Co(MoO_4_)_3_ où les dimères *M*_2_O_10_ (*M* = Co/Na) sont orientés perpendiculairement les uns aux autres (Fig. 3 ▸).

## Synthèse et cristallisation   

**Figure 7:**
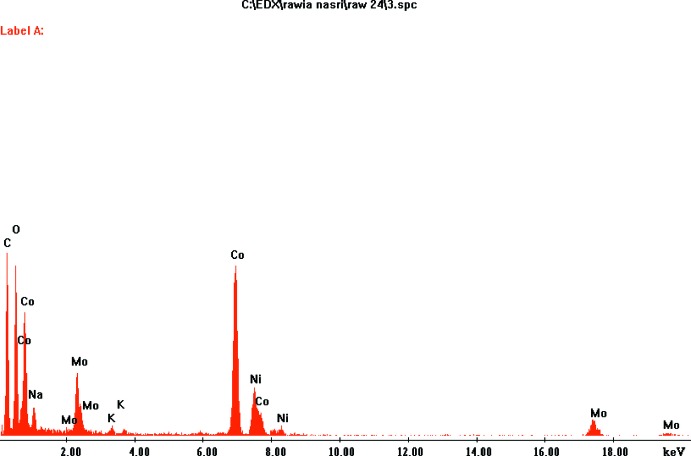
Analyse MET d’un cristal choisi de K_0.4_Na_3.6_Co(MoO_4_)_3_.

Les cristaux relatifs à K_0.4_Na_3.6_Co(MoO_4_)_3_ ont été obtenus par réaction à l’état solide à partir des réactifs: Na_2_CO_3_ (PROLABO, 70128), Co(NO_3_)·6H_2_O (FLUKA, 60832), K_2_CO_3_ (PROLABO, 60109) et (NH_4_)_2_Mo_4_O_13_ (FLUKA, 69858) pris dans les proportions telque les rapports Na:K:Co:Mo = 2:1:3:3. Après un broyage poussé dans un mortier en agate, le mélange a été mis dans un creuset en porcelaine préchauffé à l’air à 673 K pendant 12 heures en vue d’éliminer les composés volatils. Il est ensuite porté jusqu’à une température de synthèse proche de celle de la fusion à 963 K. Le mélange est maintenue à cette température pendant deux semaines pour favoriser la germination et la croissance des cristaux. Par la suite, il a subi en premier lieu un refroidissement lent (5°/jour) jusqu’à 910 K puis rapide (50°/h) jusqu’à la température ambiante. Des cristaux de couleur violet, de taille suffisante pour les mesures des intensités, ont été séparés du flux par l’eau chaude. Une analyse qualitative au MET de marque FEI et de type *QUANTA* 200 confirme la présence des éléments chimiques attendus: K, Na, Co, Mo et l’oxygène (Fig. 7 ▸).

## Affinement   

Détails de données crystallines, collection de données et affinement sont résumées dans le tableau 1 ▸. Des contraintes EADP et EXYZ pour les couples K4/Na4 conduit à des ellipsoïdes bien définis. Les densités d’électrons residuelles sont acceptables et sont situées respectivements à 0,80 Å de Mo2 et à 0,99 Å de Mo1.

## Supplementary Material

Crystal structure: contains datablock(s) I. DOI: 10.1107/S2056989014025894/br2244sup1.cif

Structure factors: contains datablock(s) I. DOI: 10.1107/S2056989014025894/br2244Isup2.hkl

CCDC reference: 1036131

Additional supporting information:  crystallographic information; 3D view; checkCIF report

## Figures and Tables

**Table 1:** Dtails exprimentaux

Donnes crystallines	
Formule chimique	K_0.4_Na_3.6_Co(MoO_4_)_3_
*M*_r_	637,15
Systme cristallin, groupe d’espace	Monoclinique, *C*2/*c*
Temprature (K)	298
*a*, *b*, *c* ()	12,8054(8), 13,5328(9), 7,1888(6)
()	112,239(8)
*V* (^3^)	1153,10(14)
*Z*	4
Type de rayonnement	Mo *K*
(mm^1^)	4.94
Taille des cristaux (mm)	0,22 0,16 0,12

Collection de donnes	
Diffractomtre	EnrafNonius CAD-4
Correction d’absorption	scan (North *et al.*, 1968 ▸)
*T*_min_, *T*_max_	0,557, 0,607
Nombre de rflexions mesures, indpendantes et observes [*I* > 2(*I*)]	2813, 1252, 1092
*R*_int_	0,029
(sin /)_max_ (^1^)	0,638

Affinement	
*R*[*F* ^2^ > 2(*F* ^2^)], *wR*(*F* ^2^), *S*	0,022, 0,056, 1,05
Nombre de rflexions	1252
Nombre de paramtres	99
Nombre de restraints	1
_max_, _min_ (e ^3^)	0,47, 0,43

Programmes informatiques: *CAD-4 EXPRESS* (Duisenberg, 1992 ▸; Macek Yordanov, 1992 ▸), *XCAD4* (Harms Wocadlo, 1995 ▸), *SHELXS97* et *SHELXL97* (Sheldrick, 2008 ▸), *DIAMOND* (Brandenburg Putz, 1999 ▸) et *WinGX* (Farrugia, 2012 ▸).

## supplementary crystallographic information

### Crystal data

**Table:**

K_0.4_Na_3.6_Co(MoO_4_)_3_	*F*(000) = 1185
*M**_r_* = 637.15	*D*_x_ = 3.670 Mg m^−^^3^
Monoclinic, *C*2/*c*	Mo *K*α radiation, λ = 0.71073 Å
Hall symbol: -C 2yc	Cell parameters from 25 reflections
*a* = 12.8054 (8) Å	θ = 10–15°
*b* = 13.5328 (9) Å	µ = 4.94 mm^−^^1^
*c* = 7.1888 (6) Å	*T* = 298 K
β = 112.239 (8)°	Prism, purple
*V* = 1153.10 (14) Å^3^	0.22 × 0.16 × 0.12 mm
*Z* = 4	

### Data collection

**Table:**

Enraf–Nonius CAD-4 diffractometer	1092 reflections with *I* > 2σ(*I*)
Radiation source: fine-focus sealed tube	*R*_int_ = 0.029
Graphite monochromator	θ_max_ = 27.0°, θ_min_ = 2.3°
ω/2θ scans	*h* = −16→13
Absorption correction: ψ scan (North *et al.*, 1968)	*k* = −2→17
*T*_min_ = 0.557, *T*_max_ = 0.607	*l* = −9→9
2813 measured reflections	2 standard reflections every 120 min
1252 independent reflections	intensity decay: 1.4%

### Refinement

**Table:**

Refinement on *F*^2^	Primary atom site location: structure-invariant direct methods
Least-squares matrix: full	Secondary atom site location: difference Fourier map
*R*[*F*^2^ > 2σ(*F*^2^)] = 0.022	*w* = 1/[σ^2^(*F*_o_^2^) + (0.0256*P*)^2^ + 0.2937*P*] where *P* = (*F*_o_^2^ + 2*F*_c_^2^)/3
*wR*(*F*^2^) = 0.056	(Δ/σ)_max_ < 0.001
*S* = 1.05	Δρ_max_ = 0.47 e Å^−^^3^
1252 reflections	Δρ_min_ = −0.43 e Å^−^^3^
99 parameters	Extinction correction: *SHELXL97* (Sheldrick, 2008), Fc^*^=kFc[1+0.001xFc^2^λ^3^/sin(2θ)]^-1/4^
1 restraint	Extinction coefficient: 0.00215 (15)

### Special details

**Table:**

Geometry. All e.s.d.'s (except the e.s.d. in the dihedral angle between two l.s. planes) are estimated using the full covariance matrix. The cell e.s.d.'s are taken into account individually in the estimation of e.s.d.'s in distances, angles and torsion angles; correlations between e.s.d.'s in cell parameters are only used when they are defined by crystal symmetry. An approximate (isotropic) treatment of cell e.s.d.'s is used for estimating e.s.d.'s involving l.s. planes.
Refinement. Refinement of *F*^2^ against ALL reflections. The weighted *R*-factor *wR* and goodness of fit *S* are based on *F*^2^, conventional *R*-factors *R* are based on *F*, with *F* set to zero for negative *F*^2^. The threshold expression of *F*^2^ > σ(*F*^2^) is used only for calculating *R*-factors(gt) *etc*. and is not relevant to the choice of reflections for refinement. *R*-factors based on *F*^2^ are statistically about twice as large as those based on *F*, and *R*- factors based on ALL data will be even larger.

### Fractional atomic coordinates and isotropic or equivalent isotropic displacement parameters (Å^2^)

**Table:**

	*x*	*y*	*z*	*U*_iso_*/*U*_eq_	Occ. (<1)
Mo1	0.5000	0.21535 (3)	0.2500	0.02386 (14)	
Mo2	0.73366 (2)	0.60949 (2)	0.62347 (4)	0.02137 (12)	
Co1	0.71350 (6)	0.33825 (6)	0.62440 (10)	0.01883 (17)	0.50
Na1	0.71350 (6)	0.33825 (6)	0.62440 (10)	0.01883 (17)	0.50
Na2	0.5000	0.23393 (16)	0.7500	0.0245 (4)	
Na3	0.5000	0.0000	0.5000	0.0358 (5)	
K4	0.5000	0.48817 (18)	0.2500	0.0434 (8)	0.402 (16)
Na4	0.5000	0.48817 (18)	0.2500	0.0434 (8)	0.597 (18)
O1	0.6713 (2)	0.6703 (2)	0.3919 (4)	0.0310 (6)	
O2	0.7192 (2)	0.6791 (2)	0.8226 (4)	0.0367 (7)	
O3	0.5413 (2)	0.2891 (2)	0.4691 (4)	0.0291 (6)	
O4	0.6072 (3)	0.1336 (2)	0.2506 (5)	0.0461 (8)	
O5	0.8768 (2)	0.5878 (2)	0.6818 (4)	0.0329 (6)	
O6	0.6623 (3)	0.4960 (2)	0.6026 (5)	0.0438 (8)	

### Atomic displacement parameters (Å^2^)

**Table:**

	*U*^11^	*U*^22^	*U*^33^	*U*^12^	*U*^13^	*U*^23^
Mo1	0.0371 (3)	0.0170 (2)	0.0127 (2)	0.000	0.00406 (17)	0.000
Mo2	0.02105 (18)	0.0256 (2)	0.01562 (17)	−0.00171 (12)	0.00482 (12)	0.00182 (11)
Co1	0.0207 (3)	0.0214 (4)	0.0145 (3)	0.0035 (3)	0.0068 (3)	0.0003 (3)
Na1	0.0207 (3)	0.0214 (4)	0.0145 (3)	0.0035 (3)	0.0068 (3)	0.0003 (3)
Na2	0.0237 (9)	0.0250 (11)	0.0276 (10)	0.000	0.0129 (8)	0.000
Na3	0.0478 (14)	0.0220 (11)	0.0246 (11)	−0.0008 (11)	−0.0011 (10)	0.0008 (9)
K4	0.0238 (10)	0.0710 (17)	0.0308 (10)	0.000	0.0051 (7)	0.000
Na4	0.0238 (10)	0.0710 (17)	0.0308 (10)	0.000	0.0051 (7)	0.000
O1	0.0330 (14)	0.0347 (16)	0.0204 (12)	0.0102 (13)	0.0046 (11)	0.0015 (12)
O2	0.0410 (16)	0.0462 (18)	0.0242 (14)	0.0009 (14)	0.0139 (12)	0.0005 (13)
O3	0.0328 (13)	0.0357 (15)	0.0189 (12)	−0.0025 (12)	0.0098 (10)	−0.0051 (11)
O4	0.058 (2)	0.0285 (16)	0.0375 (16)	0.0091 (15)	0.0012 (15)	−0.0093 (14)
O5	0.0270 (14)	0.0289 (15)	0.0400 (16)	0.0025 (12)	0.0096 (12)	0.0084 (13)
O6	0.0439 (18)	0.0396 (18)	0.0457 (18)	−0.0114 (15)	0.0145 (14)	0.0076 (15)

### Geometric parameters (Å, º)

**Table:**

Mo1—O4^i^	1.762 (3)	Na2—O1^vii^	2.416 (3)
Mo1—O4	1.762 (3)	Na2—O5^viii^	2.461 (3)
Mo1—O3	1.768 (2)	Na2—O5^v^	2.461 (3)
Mo1—O3^i^	1.768 (2)	Na3—O5^ix^	2.530 (3)
Mo2—O5	1.744 (3)	Na3—O5^v^	2.530 (3)
Mo2—O1	1.755 (3)	Na3—O4^i^	2.554 (3)
Mo2—O6	1.764 (3)	Na3—O4^x^	2.554 (3)
Mo2—O2	1.781 (3)	Na3—O5^ii^	2.677 (3)
Co1—O4^ii^	2.159 (4)	Na3—O5^viii^	2.677 (3)
Co1—O3	2.166 (3)	K4—O6	2.602 (3)
Co1—O1^iii^	2.188 (3)	K4—O6^i^	2.602 (3)
Co1—O2^iv^	2.211 (3)	K4—O6^iv^	2.675 (3)
Co1—O6	2.222 (3)	K4—O6^vii^	2.675 (3)
Co1—O2^v^	2.298 (3)	K4—O3^i^	3.064 (3)
Na2—O3^vi^	2.392 (3)	K4—O3	3.064 (3)
Na2—O3	2.392 (3)	K4—O1	3.199 (4)
Na2—O1^iii^	2.416 (3)	K4—O1^i^	3.199 (4)
			
O4^i^—Mo1—O4	102.2 (2)	O3—Co1—O1^iii^	83.96 (10)
O4^i^—Mo1—O3	109.06 (14)	O4^ii^—Co1—O2^iv^	90.15 (12)
O4—Mo1—O3	112.50 (13)	O3—Co1—O2^iv^	82.34 (11)
O4^i^—Mo1—O3^i^	112.50 (13)	O1^iii^—Co1—O2^iv^	165.35 (11)
O4—Mo1—O3^i^	109.06 (14)	O4^ii^—Co1—O6	95.65 (12)
O3—Mo1—O3^i^	111.26 (18)	O3—Co1—O6	92.46 (11)
O5—Mo2—O1	111.51 (13)	O1^iii^—Co1—O6	87.03 (12)
O5—Mo2—O6	109.70 (15)	O2^iv^—Co1—O6	98.70 (12)
O1—Mo2—O6	107.00 (15)	O4^ii^—Co1—O2^v^	79.83 (11)
O5—Mo2—O2	108.41 (14)	O3—Co1—O2^v^	92.45 (11)
O1—Mo2—O2	111.48 (13)	O1^iii^—Co1—O2^v^	90.38 (11)
O6—Mo2—O2	108.69 (15)	O2^iv^—Co1—O2^v^	85.05 (11)
O4^ii^—Co1—O3	169.69 (11)	O6—Co1—O2^v^	174.18 (11)
O4^ii^—Co1—O1^iii^	102.76 (12)		

Symmetry codes: (i) −*x*+1, *y*, −*z*+1/2; (ii) −*x*+3/2, −*y*+1/2, −*z*+1; (iii) *x*, −*y*+1, *z*+1/2; (iv) *x*, −*y*+1, *z*−1/2; (v) −*x*+3/2, *y*−1/2, −*z*+3/2; (vi) −*x*+1, *y*, −*z*+3/2; (vii) −*x*+1, −*y*+1, −*z*+1; (viii) *x*−1/2, *y*−1/2, *z*; (ix) *x*−1/2, −*y*+1/2, *z*−1/2; (x) *x*, −*y*, *z*+1/2.
